# Effects of Sodium Silicate Complex against Hemorrhagic Activities Induced by *Protobothrops mucrosquamatus* Venom

**DOI:** 10.3390/toxins13010059

**Published:** 2021-01-14

**Authors:** Yen-Chia Chen, Tse-Yao Wang, Yu-Kai Huang, Kun-Che Chang, Min-Hui Chen, Chien-Chun Liu, Kuei-Lin Liu, Ya-Han Yang, David Hung-Tsang Yen, Ju-Sing Fan

**Affiliations:** 1 Emergency Department, Taipei Veterans General Hospital, Taipei 11217, Taiwan; ycchen4@gmail.com (Y.-C.C.); tseyao85@gmail.com (T.-Y.W.); hjyen@vghtpe.gov.tw (D.H.-T.Y.); 2 Department of Emergency Medicine, School of Medicine, National Yang-Ming University, Taipei 11221, Taiwan; 3 Department of Emergency Medicine, School of Medicine, National Defense Medical Center, Taipei 11490, Taiwan; 4 Graduate Institute of Medicine, College of Medicine, Kaohsiung Medical University, Kaohsiung 80708, Taiwan; yukaih@gmail.com; 5 Division of Neurosurgery, Department of Surgery, Kaohsiung Medical University Hospital, Kaohsiung 80708, Taiwan; 6 Department of Surgery, Kaohsiung Municipal Ta-Tung Hospital, Kaohsiung 80145, Taiwan; 7 Department of Ophthalmology, Louis J. Fox Center for Vision Restoration, University of Pittsburgh School of Medicine, Pittsburgh, PA 15213, USA; kcchang@pitt.edu; 8 Spencer Center for Vision Research, Byers Eye Institute, School of Medicine, Stanford University, Palo Alto, CA 94304, USA; 9 Enkang Clinic, 3F, 88, Baozhong Rd., Xindian Dist, New Taipei 23144, Taiwan; chen.minhui@gmail.com; 10 Molecular Medicine Research Center, Chang Gung University, Taoyuan 33302, Taiwan; chienchunliu1016@gmail.com; 11 Faculty of Biotechnology and Laboratory Science in Medicine, School of Medical Technology and Engineering, National Yang-Ming University, Taipei 11221, Taiwan; jerry860822@gmail.com; 12 School of Medicine, College of Medicine, Chang Gung University, Taoyuan 33302, Taiwan; yangyahan8246@gmail.com

**Keywords:** snake venom, snakebite, hemorrhage, *Protobothrops mucrosquamatus*

## Abstract

*Protobothrops mucrosquamatus* poses a serious medical threat to humans in Southern and Southeastern Asia. Hemorrhage is one of the conspicuous toxicities related to the pathology of *P. mucrosquamatus* envenoming. Previous in vitro and in vivo studies showed that a silica-derived reagent, sodium silicate complex (SSC), was able to neutralize hemorrhagic and proteolytic activities induced by pit viper venoms, including *Crotalus atrox*, *Agkistrodon* *contortrix contortrix* and *Agkistrodon piscivorus leucostoma*. In this study, we validated that SSC could neutralize enzymatic and toxic effects caused by the venom of *P. mucrosquamatus*. We found that SSC inhibited the hemolytic and proteolytic activities induced by *P. mucrosquamatus* venom in vitro. In addition, we demonstrated that SSC could block intradermal hemorrhage caused by *P. mucrosquamatus* venom in a mouse model. Finally, SSC could neutralize lethal effects of *P. mucrosquamatus* venom in the mice. Therefore, SSC is a candidate for further development as a potential onsite first-aid treatment for *P. mucrosquamatus* envenoming.

## 1. Introduction

Envenoming by venomous snakes poses a serious and challenging global health issue. Kasturiratne et al. [1] estimated that at least 421,000 envenomings and 20,000 deaths occur each year resulting from snakebites. The highest burden exists in South Asia, Southeastern Asia, and sub-Saharan Africa. The World Health Organization (WHO) recently included snake envenoming as a category A disease in its list of Neglected Tropical Diseases [2]. The mainstay therapy of snake envenoming is the intravenous administration of animal-derived antivenoms, including IgG, fragments F(ab) and F(ab′)2. Although antivenom therapy is largely successful in reducing the mortality associated with snake envenoming, the efficacy of antivenom administration against local symptoms has been limited due to rapid development of damage at the bite sites [3,4,5].

Hemotoxic venom is composed of proteolytic enzymes and mixed compounds. Among the mixtures, snake venom metalloproteinases (SVMP), phospholipases A_2_ (PLA_2_s), and serine proteases mainly account for systemic and local toxicities, such as hemorrhage, allergy, edema, necrosis, and even shock via circulatory system disturbance [6,7,8,9]. To alleviate local toxicity caused by venom, some experimental studies [4,10,11] reported a novel therapy for the early stage of envenomation using chelators to neutralize metals such as Ca^2+^, Mg^2+^, Zn^2+^, which are known co-factors in the active site of hemotoxic enzymes found in snake venom [12,13,14]. Although DTPA and EDTA, two well-known metal chelators, can neutralize the hemotoxic activities, they provide no significant improvement of either reducing tissue necrosis or increasing survival rate after snake envenomation [15]. In addition, EDTA is contraindicated in the presence of electrolyte imbalance, which is not uncommon after snakebite.

**Figure 1:**
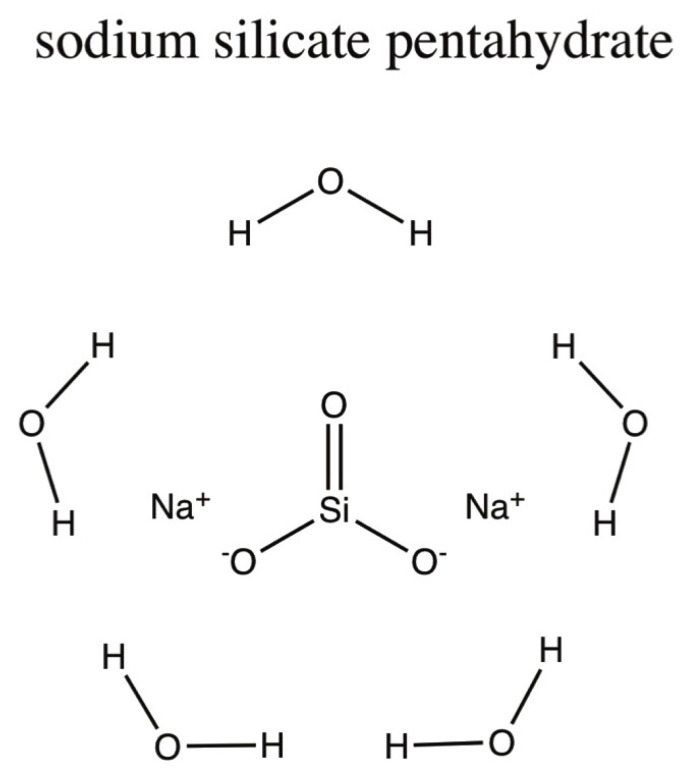
Chemical structure of sodium silicate pentahydrate (SSC).

A study from the National Natural Toxins Research Center (NNTRC, Texas A&M University-Kingsville, Kingsville, TX, USA) described a patented chemical material, sodium silicate complex (SSC), that was able to neutralize the hemotoxic activity, proteolytic activity and hyaluronidase activity in snake venoms from *Crotalus atrox*, *Agkistrodon contortrix contortrix*, and *A. piscivorus leucostoma* [16]. SSC is a modified silica complex (Figure 1) that is water-soluble, carries a pH = 13.7 without having corrosive properties, and is very stable at room temperature. A study conducted in NNTRC also demonstrated that SSC alleviates the subcutaneous hemorrhage induced by venom injection in a mouse model [16]. These findings explain the capability of SSC to block hemorrhage caused by pit viper venoms. Most importantly, SSC also shows no evidence of neither irritation on human skin nor reproductive defects in male animals [17]. Other functions of SSC, such as anti-cancer and anti-virus activities, were studied by a group of Texas State University [18,19]. These studies suggest that SSC may be a safe and beneficial product for human use.

*Protobothrops mucrosquamatus* has a wide geographic distribution including India, Bangladesh, Myanmar, Laos, Vietnam, Thailand, southern China, Taiwan, and Okinawa [20,21,22,23]. Because *P. mucrosquamatus* envenoming can inflict local effects such as pain, edema, hemorrhage, necrosis, and rare compartment syndrome as well as systemic effects including thrombocytopenia, coagulopathy, rhabdomyolysis, acute renal injuries [24], limb disabilities [22] and death [25], the World Health Organization lists *P. mucrosquamatus* among the venomous snakes of highest medical importance in Asia [26]. There are several issues related to antivenom therapy that compromise their effectiveness, including delayed administration of antivenoms in rural settings where public health services and health personnel are scarce [27] and limited effectiveness of antivenom against venom-induced hemorrhage, necrosis and severe soft tissue swelling developing into airway compromise [28]. In addition, subretinal hemorrhage may occur after snake envenoming, even administered with high doses of antivenom [9]. These evidences show that antivenom therapy may be only partially effective in the control of local tissue damage. In light of the evidence for SSC’s efficacy against snake venoms of *C. atrox*, *A. contortrix contortrix*, *A. piscivorus leucostoma,* and *Naja* subfamily species, SSC administration could be a potential onsite first-aid management to reduce the severity of hemorrhage, edema, and even tissue necrosis prior to antivenom administration in the hospital. To better understand if SSC can neutralize the toxic effects of *P. mucrosquamatus* venom, we studied the neutralizing effects of SSC on the *P. mucrosquamatus* venom in vitro and the protective effects against the intradermal hemorrhage and lethality induced by *P. mucrosquamatus* venom in vivo.

## 2. Results

### 2.1. In Vitro Evaluation of SSC against Hemolytic and Proteolytic Activities Induced by P. mucrosquamatus Venom

**Figure 2:**
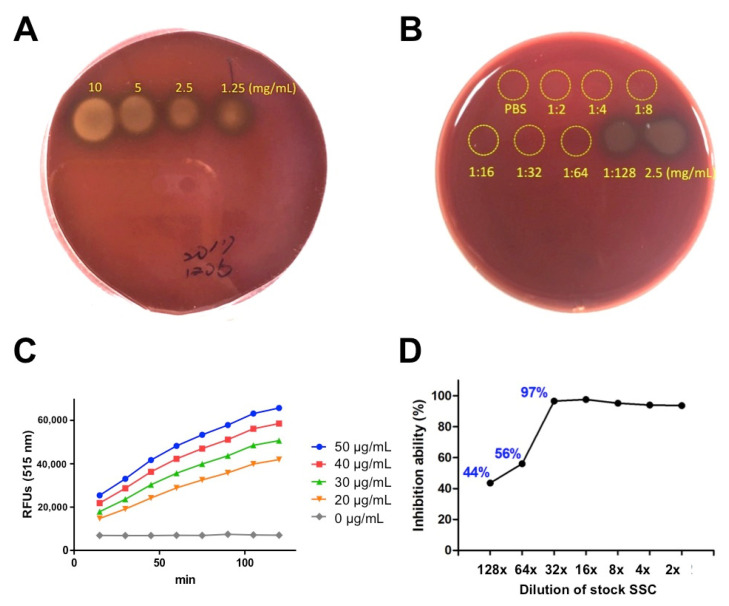
Hemolytic and proteolytic activities of *P. mucrosquamatus* venom in different concentrations of SSC. (**A**) The hemolytic circles were induced by different concentrations (10, 5, 2.5, and 1.25 mg/mL) of *P. mucrosquamatus* venom in blood agar. (**B**) Equal amount of venom (2.5 mg/mL) was pre-incubated with different dilutions. 1:2 indicates 2-fold dilution of stock SSC (130 mg/mL) and so on. PBS was used for negative control. (**C**) The proteolytic activity of different concentrations of *P. mucrosquamatus* venom was assessed by the DQ-gelatin assay. (**D**) Equal amounts of venom (50 μg/mL) were pre-incubated with different dilutions (2–128×) of stock SSC (130 mg/mL) and subjected to evaluation of the inhibitory activity. PBS and venom were used for negative and positive controls, respectively.

In a previous study, SSC was able to neutralize hemorrhagic and proteolytic activities of venom from *C. atrox*, *A. c. contortrix* and *A. p. leucostoma* in vitro and in vivo [16]. Here, we further investigate whether SSC also inhibits the hemolytic activities caused by *P. mucrosquamatus* venom. Using blood agar, we tested serial concentrations of *P. mucrosquamatus* venom (Figure 2A) and found that *P. mucrosquamatus* venom pre-incubated with SSC significantly reduced the hemolytic activity and the working dilution of SSC against *P. mucrosquamatus* venom can be as low as 64-fold dilution of stock SSC (2.03 mg/mL), which no hemolytic zone was observed (Figure 2B). We next examined the proteolytic activity of *P. mucrosquamatus* venom using the gelatinase assay kit. We observed that *P. mucrosquamatus* venom showed enzymatic digestion of fluorescein-labeled DQ gelatin in both time- and dose-dependent manners, indicating the proteolytic activity of *P. mucrosquamatus* venom (Figure 2C). Data showed that a 128-fold dilution of stock SSC (1.02 mg/mL) inhibited 44% of proteolytic activity and a 32-fold dilution of SSC (4.06 mg/mL) inhibited 97% of proteolytic activity, respectively (Figure 2D). These in vitro data indicated that SSC is a strong blocker of both hemolytic and proteolytic components in the *P. mucrosquamatus* venom and that a 64-fold dilution of SSC (2.03 mg/mL) is an optimal dose for following in vivo study.

### 2.2. The Hemorrhagic and Proteolytic Features of P. mucrosquamatus Venom Was Blocked by SCC Pre-Mixture

**Figure 3:**
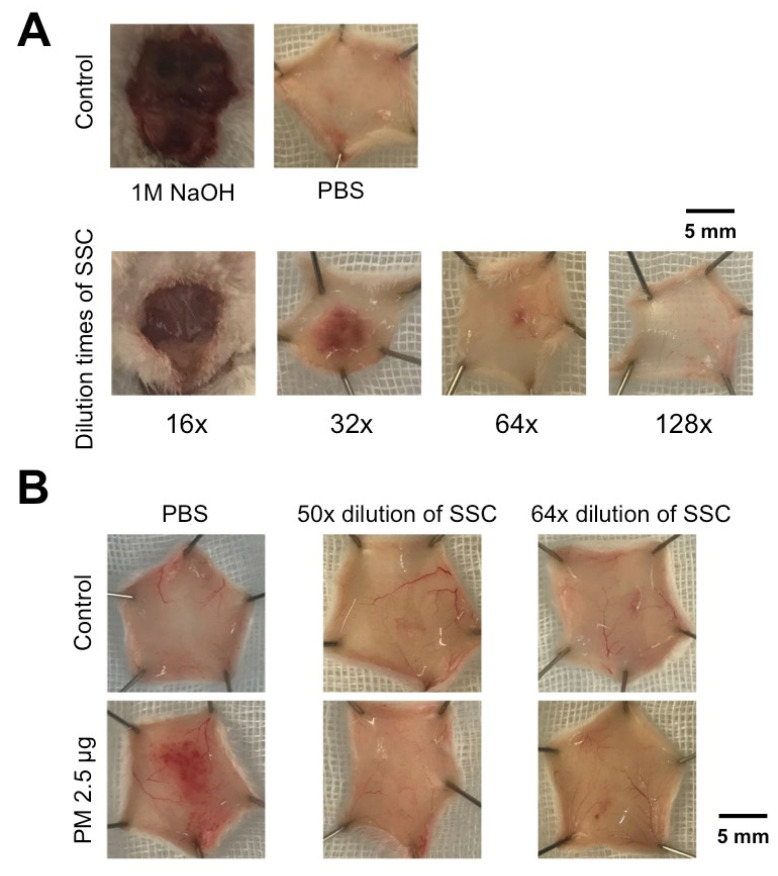
Intradermal test of SSC and SSC/venom mixture. (**A**) The safe activity of SSC was tested in various dilutions (16 to 128× indicates 16 to 128-fold) by intradermal injection. 1M NaOH and PBS were used for positive and negative controls, respectively. (**B**) Mixture of *P. mucrosquamatus* venom (2.5 μg) and diluted SSC (2.60 mg/mL and 2.03 mg/mL for 50-fold and 64-fold dilution, respectively) was subjected to evaluate the anti-hemorrhagic activity by intradermal injection.

To apply SSC in vivo, we first performed a subcutaneous test with different dilutions of SSC (8–128×) as well as positive (NaOH) and negative (PBS) controls using intradermal injection in a mouse model. It showed that 16–32× dilution of SSC injection may cause intradermal injury, but 64× dilution didn’t. We therefore selected the 64× dilution of SSC (2.03 mg/mL) for anti-hemorrhagic treatment (Figure 3A). To further test the inhibitory effect of SSC on subcutaneous hemorrhage induced by *P. mucrosquamatus* venom, we intradermally injected the SSC-PM mixture and found that either the 50× or 64× dilution of SSC (2.60 or 2.03 mg/mL) was able to block the hemorrhagic effect in vivo (Figure 3B). Compared to 50× dilution of SSC, we found that 64× dilution of SCC not only had similar inhibitory effect against venom but also caused less intradermal injury. We therefore chose 64× dilution of SSC for the following studies. These data suggest that 64× dilution (2.03 mg/mL) is a safe and effective concentration of SSC in vivo.

### 2.3. In Vivo Evaluation of Post-Treatment SSC against Intradermal Hemorrhage Induced by P. mucrosquamatus Venom

**Figure 4:**
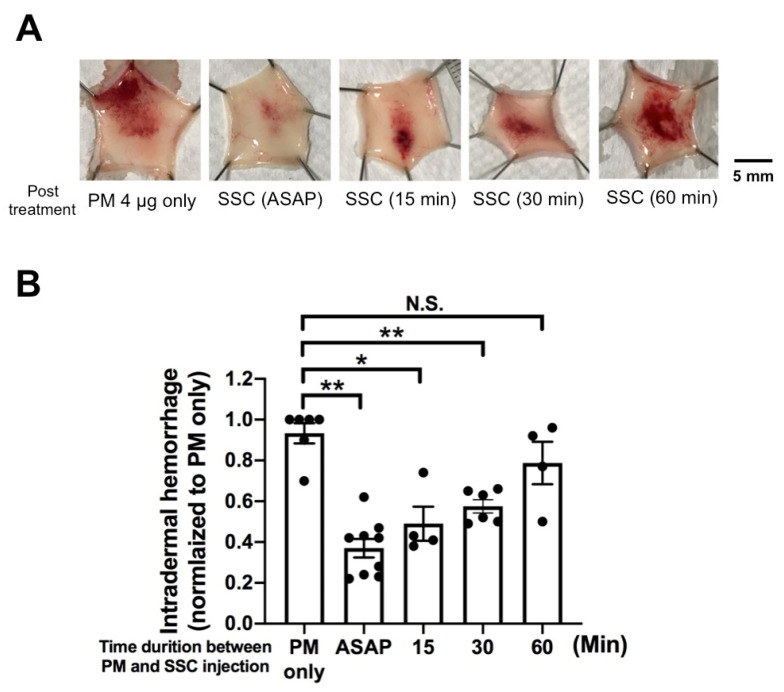
Post-treatment of SSC on venom-induced intradermal hemorrhage. (**A**) 64-fold (64×) diluted SSC (2.03 mg/mL) was applied to *P. mucrosquamatus* venom (4 μg) injected wound as soon as possible (ASAP) or at variable time points. (**B**) Statistical bar chart indicating the severity of hemorrhage. ASPS indicates the interval of 1 min between venom and SSC injection. *n* ≥ 4 for each group. * *p* < 0.05, ** *p* < 0.01, N.S., no significant difference. One-way ANOVA post-hoc *t*-test with Tukey correction. Means ± SEMs shown.

In clinical practice, patients are treated after snake envenoming. To simulate such a scenario, we further conducted a post treatment study (where injection of venom into the dermal layer of the mouse is followed by SSC injection near the lesion at a later time point) to reveal the relationship between the time delay of treatment and the anti-hemorrhagic effect (Figure 4A). We observed that SSC significantly reduced about 70% and 50% of the intradermal hemorrhage when the treatment is given as soon as possible and within 30 min, respectively (Figure 4B). However, we found no significant improvement of hemorrhage if SSC is administered after 60 min. These data suggest that SSC should be given within 30 min post bite to maximize therapeutic efficacy.

### 2.4. In Vivo Evaluation of Lethality Neutralization Effect of SSC against P. mucrosquamatus Venom

**Figure 5:**
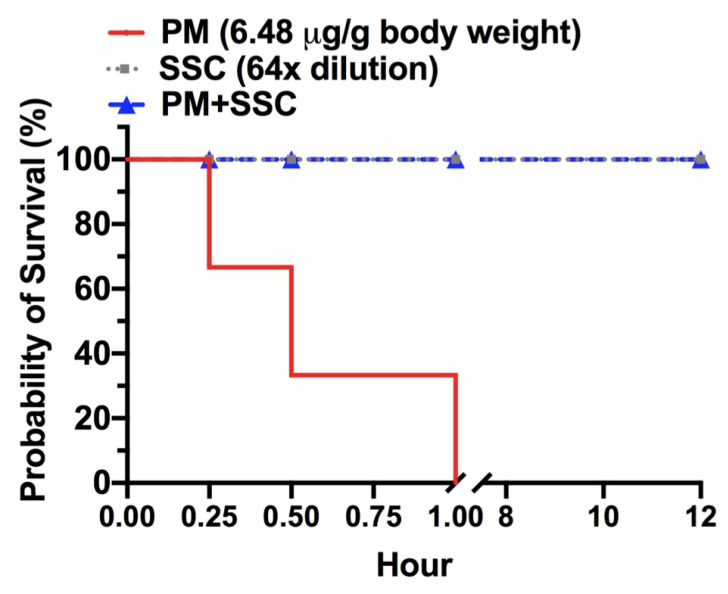
Effect of SSC on venom-induced lethality. Post-treatment of 64-fold (64×) diluted SSC (2.03 mg/mL) prevents death of *P. mucrosquamatus* venom (6.48 μg/g of body weight) injected mice for 12 h. *n* = 3 for each group.

To understand if SSC can neutralize the lethal effect of *P. mucrosquamatus* venom, we conducted a survival study in mice. After intraperitoneal injection of 3-fold LD_50_ of *P. mucrosquamatus* venom to mice, we observed 100% mortality within an hour (Figure 5). To the contrary, the venom with 64-fold diluted SSC pre-treatment allowed animals to survive the observation period of 12 h after the intraperitoneal injection. Then, all survival mice were sacrificed by CO_2_ inhalation to meet the principles of the 3Rs (Replacement, Reduction and Refinement) in animal study. These data suggested SSC was effective in neutralizing lethal components in the *P. mucrosquamatus* venom.

## 3. Discussion

In this study, we found that SSC in vitro neutralized the hemolytic and proteolytic activities induced by the *P. mucrosquamatus* venom. We also found that SSC showed a promising effect against the hemorrhagic activity caused by *P. mucrosquamatus* venom in a mouse model of dermal hemorrhage. Furthermore, we observed that SSC administration increased probability of survival in mice treated with *P. mucrosquamatus* venom. *P. mucrosquamatus* venom consists of a complex mixture of proteins and peptides, mainly SVMPs (43%), PLA_2_s (22.5%), snake venom serine proteases (SVSPs, 10.4%) and peptides (6.9%) [29,30,31,32]. SVMPs participate in the hemorrhagic process by inducing proteolytic degradation of extracellular matrix components that are involved in the maintenance of the structural and functional integrity of capillaries, resulting in the disruption of microvasculature and causing edema and hemorrhage [32]. SSC is a silicon-derived material conjugated with sodium, and based on its chemical structure, we hypothesized the SSC alleviates hemorrhage by inhibiting SVMPs. It remains unclear if the anti-hemorrhagic activity of SSC is similar to other metal (Zn^2+^ or Ca^2+^) chelator mechanisms of SVMPs reported in previous studies [12,13,14]. It is assumed that the inhibition of SVMPs in an early phase of envenoming may be a promising alternative therapy. In this study we did not validate SSC’s effect on SVMPs and such experiment would be an interesting direction in the future. Wound infection may occur after *P. mucrosquamatus* envenoming [33]. Many metal ions such as iron and zinc have been reported as bactericides [34]. Sodium [35] and SSC [36] have also been shown to inhibit bacteria growth. This suggests that SSC may not only attenuate the venom-induced hemorrhage, but also prevent the bacterial infection complicating *P. mucrosquamatus* envenoming.

Our data revealed that SSC effectively reduced the venom-induced hemorrhage if administered within 30 min after envenoming in the mouse model. Moreover, the sooner SSC was delivered, the better the anti-hemorrhagic effects were (Figure 4). However, we did not observe any significant effects at 60 min. A possible explanation is that *P. mucrosquamatus* venom destroyed the structural and functional integrity of capillaries to cause maximal hemorrhage by 60 min, and thus SSC was not able to stop the bleeding via its neutralizing capability. We found that SSC treatment prevented lethality of the *P. mucrosquamatus* venom in mice. PLA_2_s of *P. mucrosquamatus* are known to have diverse biological effects, including myotoxicity (promutoxin) [37,38], hemorrhage and cytotoxicity [39], and pre-synaptic neurotoxicity (trimucrotoxin) [40,41]. Since neurotoxic and myotoxic PLA_2_ have been reported to cause mortality in mice [32], it is possible that SSC can block PLA_2_s in *P. mucrosquamatus* venom. In the clinical setting, these toxins may cause soft tissue hemorrhage and necrosis after *P. mucrosquamatus* envenoming. About 5% of patients subsequently developed long-term limb disabilities after suffering *P. mucrosquamatus* bite wounds [23]. In this study, though we did not study the capability of SSC against other neurotoxic and cytotoxic components derived from *P. mucrosquamatus* venom, we speculated that SSC probably inhibits these enzymes or peptides to nullify the lethal effects. In addition, some PLA_2_s may play a role in developing edematogenic activity, mast cell degranulation and inflammation [30,42], while SVSPs are coagulotoxins that may cause coagulopathy after *P. mucrosquamatus* envenoming [43]. Thus, further studies are needed to explore whether SSC excises its capability to block PLA_2_s and SVSPs.

Although we demonstrated promising results of SSC against hemolytic and proteolytic activities of *P. mucrosquamatus* venom in vitro as well as the intradermal hemorrhage in vivo, there are some limitations in the present study. First, real-world *P. mucrosquamatus* bites may penetrate deep into the skin and muscle, but we do not know the extent to which SSC could infiltrate into the deep soft tissue or muscle to neutralize the *P. mucrosquamatus* venom. This study was conducted using the intradermal injection of *P. mucrosquamatus* venom followed by SSC intradermal injection. Thus, SSC treatment for different depths of venom penetration will be another focus of research. Second, in this experimental study, the dose of *P. mucrosquamatus* venom injection is small and consistent. Whether SSC can effectively neutralize higher doses of *P. mucrosquamatus* venom remains unclear. Third, there is no comparison to other treatments (e.g., antivenom) or other administration routes in this study. It would be better to conduct a study compared with a conventional anti-hemorrhagic treatment and different administration routes in the future.

## 4. Conclusions

Our study demonstrates SSC to be effective against *P. mucrosquamatus* venom in vitro and in vivo. SSC could be considered for further development as onsite first-aid management due to its easy accessibility and potential effectiveness. Unlike protein-based antivenom agents, SSC is thermo-stable that can be preserved in room temperature and purchased easily from online shops. In addition, previous studies suggested that 2–4 vials are generally recommended to treat *P. mucrosquamatus* envenoming [44,45]. A recent study reported that the median dose of antivenom to treat *P. mucrosquamatus* envenoming is 5.5 vials [33]. Given that the high cost of antivenom production and risk of allergic reactions associated with antivenom administration, onsite first-aid SSC application to the snakebite wound in the prehospital setting might be expected to alleviate the local toxicities of venom and thus reduce the total burden of antivenom in subsequent treatment.

## 5. Materials and Methods

### 5.1. Snake Venom and Chemical Materials

The crude venom powder (pool mixture of age and gender) of P. mucrosquamatus was obtained from the Centers for Disease Control, R.O.C. (Taiwan). Venom was dissolved in PBS before use. Solutions of NaOH (1M) and PBS (pH = 7.4) were purchased from Sigma-Aldrich (St. Louis, MO, USA). Liquid SSC (130 mg/mL) was obtained from Cisne Enterprises Inc. (Odessa, TX, USA), and stored at 4 °C until used. All use of animals was approved by the Institutional Animal Care and Use Committee (IACUC, #2018-256) of the Taipei Veterans General Hospital and Chang Gung University. Adult male ICR mice (25–30 g) for this study were obtained from BioLASCO, Taiwan Co., Ltd. (Taipei City, Taiwan). Mice were housed at ~25 °C and photoperiod (12 h light, 12 h dark) standard, filter-top plastic rodent cages with regular diet.

### 5.2. Blood Agar Assay

The self-made sheep blood agar (from a local farm) was produced following the protocol described by Microbiology Info.com. The blood agar assay was used for measuring the hemolytic activity caused by *P. mucrosquamatus* venom. Different concentrations (10, 5, 2.5, and 1.25 mg/mL) of *P. mucrosquamatus* venom (4 μL) were dropped on the blood agar. For inhibitory observation, an equal amount of diluted SSC was pre-incubated with venom and immediately dropped on the blood agar. To conduct the blood agar assay, the blood agar plate was held up to a light source to observe the area of hemolytic reaction with the light coming from the behind. The formation of the hemolytic zone was observed after 16 h incubation.

### 5.3. Fluorescence-Labeled Gelatin Substrate Assay

The EnzCheck Gelatinase Assay Kit (Thermofisher Scientific, Waltham, MA, USA) was used for SVMP activity measurement, according to the provided protocol. Briefly, *P. mucrosquamatus* venom samples were mixed with a fluorescently labeled gelatin substrate. Samples with or without SSC were incubated for 2 h at 37 °C, and the fluorescence was measured with a SpectraMax M5 microplate reader (Molecular Devices, San Jose, CA, USA) at an excitation wavelength of 495 nm and an emission wavelength of 515 nm.

### 5.4. Hemorrhagic Mouse Model

SSC neutralization ability against *P. mucrosquamatus* venom-induced hemorrhage was evaluated in this study. The definition of minimum hemorrhagic dose (MHD) is amount of *P. mucrosquamatus* venom that induces a hemorrhagic spot of 10 mm diameter in the mouse skin 2 h after injection [32]. The MHD (2.5 μg) of *P. mucrosquamatus* venom dissolved in 100 μL PBS was mixed with 50 μL of diluted SSC (2.06 mg/mL), while 50 μL of normal saline solution and diluted SSC were prepared as negative and SSC safety control. Samples with or without SSC were incubated for 30 min at 37 °C. For the animal experiment, name of the ethics approval board is Institutional Animal Care and Use Committee (IACUC) of Taipei Veterans General Hospital; project identification code is 2018-256; date of approval is 29 January 2019. A group of 3 lightly anesthetized mice were intradermally injected with a mixture of venom and SSC in the dorsal skin. Animals were sacrificed by CO_2_ inhalation 2 h post-injection. The dorsal skin was then removed and the hemorrhagic lesion in the inner side of the skin was measured. In the time-delay neutralization test, groups of 3 lightly anesthetized mice were intradermally injected with 1.6-fold MHD of venom (4 μg), then intradermally injected with diluted SSC at the same locus at 1, 15, 30, 60 min post venom injection, respectively. Animals were sacrificed by CO_2_ inhalation 2 h post SSC injection. The dorsal skin was then removed and the hemorrhagic lesion on the inner side of the skin was measured.

### 5.5. Quantification of Intradermal Hemorrhage

Images were taken by Nikon COOLPIX S5100 (NIKON Corp. Tokyo, Japan) and quantified using ImageJ 1.x (National Institute of Health, Bethesda, MD, USA) to outline and measure the hemorrhagic area. Within the region of interest, average pixel intensity values for red, green, and blue were calculated using the color histogram function of ImageJ. These values were then converted to hemorrhagic units (HaU) following the protocol as previously described [46], in order to accurately represent lesion size and intensity. Briefly, RGB values were scaled and adjusted to calculate luminance according to the luminosity function. Correction factors were then applied to normalize for image scaling and average luminance. Finally, each batch of images was normalized to an internal positive control (*P. mucrosquamatus* only).

### 5.6. Envenomation and Animal Survival Assays

The lethality neutralization effect of SSC against the *P. mucrosquamatus* venom was evaluated in mice. The definition of LD_50_ is the dose of *P. mucrosquamatus* venom that induces lethality in 50% of injected mice [32]. Three groups of 3 mice were injected intraperitoneally with SSC (64-fold diluted) only or 3-fold LD_50_ (2.16 μg/g) pre-treated with or without SSC (64-fold diluted), respectively. Samples with or without SSC were incubated for 30 min at 37 °C. The survival rate was determined by the number of live mice every 30 min over a 12 h period.

### 5.7. Statistical Analysis

Data were analyzed using a one-way ANOVA with post-hoc *t*-test with Tukey correction and/or an unpaired *t*-test with *p* value of <0.05 considered significant. All statistical analyses were performed using GraphPad Prism 5 software (GraphPad software, San Diego, CA, USA).

## Data Availability

The data presented in this study are available on request from corresponding author.
